# Association of *Leptin* Gene Polymorphisms with Rheumatoid Arthritis in a Chinese Population

**DOI:** 10.1155/2020/3789319

**Published:** 2020-10-06

**Authors:** Sha-Sha Tao, Yi-Lin Dan, Guo-Cui Wu, Qin Zhang, Tian-Ping Zhang, Yin-Guang Fan, Hai-Feng Pan

**Affiliations:** ^1^Department of Epidemiology and Biostatistics, School of Public Health, Anhui Medical University, 81 Meishan Road, Hefei, Anhui, China; ^2^Anhui Provincial Laboratory of Inflammatory and Immune Diseases, 81 Meishan Road, Hefei, Anhui, China; ^3^School of Nursing, Anhui Medical University, 15 Feicui Road, Hefei, Anhui, China

## Abstract

**Background:**

Recently, increasing studies have revealed that leptin is involved in the development of rheumatoid arthritis (RA). This study is aimed at exploring the association of *leptin* gene single nucleotide polymorphisms (SNPs) with susceptibility to RA in a Chinese population.

**Methods:**

We recruited 600 RA patients and 600 healthy controls from a Chinese population and analyzed their three *leptin* SNPs (rs10244329, rs2071045, and rs2167270) using the improved Multiplex Ligase Detection Reaction (iMLDR) assays. The associations of these SNPs with clinical manifestations of RA were also analyzed. Enzyme-linked immunosorbent assay (ELISA) was performed for plasma *leptin* determination.

**Results:**

No significant difference in either allele or genotype frequencies of these three SNPs between RA patients and healthy controls was observed (all *P* > 0.05). Association between the genotype effects of dominant, recessive models was also not found (all *P* > 0.05). No significant difference in plasma *leptin* levels was detected between RA patients and controls (*P* > 0.05).

**Conclusion:**

*Leptin* gene (rs10244329, rs2071045, and rs2167270) polymorphisms are not associated with RA genetic susceptibility and its clinical features in the Chinese population.

## 1. Introduction

Rheumatoid arthritis (RA) is one type of systemic autoimmune diseases, featured by progressive destruction of the cartilage, bone, and joint eventually, which ultimately leads to a chronic, destructive inflammatory arthritis [1]. The disease destructs synovial small and large joints, for instance, fingers, elbows, shoulders, ankles, and knees [2]. RA patients usually have severe disability or early death resulting from thickening and hyperplasia of the synovial line and chronic inflammatory [3]. With a global prevalence of 1%, RA is still a serious public health problem that harms health and increases financial burden, especially in the developing countries in Asia [4–6]. Although the precise aetiology of RA remains unknown, increasing studies have pointed out that leptin takes parts in its pathogenesis [7–11].

Leptin, the precursor and most characteristic member of the adipokine family, is a 16 kD nonglycosylated cytokine-like hormone encoded by the *obese* (*ob*) gene, the murine homolog of the human *leptin* gene [12]. In recent years, numerous studies have revealed that leptin has been implicated in inflammation and autoimmunity [13–19]. Emerging evidence has indicated that leptin is involved in RA [7, 9, 20–26]. Some studies have detected increased serum levels of leptin in RA patients [27–37], while others have suggested reduced levels [38–41].

Moreover, the function of the *leptin* gene in autoimmune diseases has received much attention in recent years [42–46]. It have been revealed that *leptin* plays a crucial role in the susceptibility to multiple sclerosis (MS) [42, 43] and systemic lupus erythematosus (SLE) [44]. It also has been reported that *leptin*-deficient mice had decreased levels of TNF-*α* and IL-1*β*, a less severe arthritis induced by antigen and defective cell-mediated immunity [47]. Nevertheless, some other studies claimed that the *leptin* polymorphism was not associated with the risk of RA [48, 49]. However, the association between *leptin* polymorphisms and RA in the Chinese population has not been studied as yet.

Therefore, in this case-control study, we detected the association of *leptin* gene polymorphisms (rs10244329, rs2071045, and rs2167270) with the susceptibility to RA in a Chinese population.

## 2. Materials and Methods

### 2.1. Patients and Control Subjects

In this study, 600 RA patients were recruited from the Department of Rheumatology and Immunology at the First Affiliated Hospital of Anhui Medical University and the First Affiliated Hospital of University of Science and Technology of China. All the RA patients met the criteria of the American College of Rheumatology (ACR)/European League Against Rheumatism collaborative initiative (EULAR) revised in 2010 [50]. And 600 healthy controls were enlisted from the Health Examination Centre of the First Affiliated Hospital of Anhui Medical University and the Second Affiliated Hospital of Anhui Medical University. All the healthy controls should have no history of any signs or symptoms of autoimmune diseases. Patients' demographic data and clinical manifestations, including anticyclic citrullinated peptide (anti-CCP) and rheumatoid factor (RF), were collected through reviewing medical records or by questionnaire. This study was approved by the Medical Ethics Committee of Anhui Medical University, and informed consent was provided by all participants.

### 2.2. DNA Extraction and SNP Genotyping

Peripheral blood was extracted from study subjects, and the genomic DNA was obtained from the peripheral blood lymphocytes according to the standard procedures with the Wizard Genomic DNA Purification Kit (Promega, Madison, Wisconsin, USA). Quantification and concentration of DNA was determined applying agarose gel electrophoresis and using a NanoDrop 2000 Spectrophotometer (Thermo Fisher Scientific, USA). Qualified sample requirements are as follows: concentration greater than 50 ng/*μ*l, total amount greater than 600 ng, and no obvious degradation.

The genotyping of three SNPs was analyzed by applying the improved Multiplex Ligase Detection Reaction (iMLDR) assay technology and an ABI3730XL automated sequencer (Applied Biosystems, USA). Only patients with 100% genotype success rate for all three SNPs can be included in this study. Therefore, finally, we analyzed the three *leptin* SNPs (rs10244329, rs2071045, and rs2167270) in 595 RA patients and 599 normal controls.

### 2.3. Plasma Leptin Detection

In the determination of plasma leptin levels, 103 RA patients and 95 health controls were recruited. We collected 5 ml peripheral venous blood of all subjects and extracted plasma. Enzyme-linked immunosorbent assay (ELISA) kits (4Abiotech, China) were used for plasma *leptin* determination.

### 2.4. Statistical Analysis

The chi-square (*χ*^2^) or Fisher's exact test was applied to analyze whether the genotype and allele frequencies of all SNPs between RA patients and controls are different. Haplotype analysis was conducted by applying an online SHEsis software (http://analysis.bio-x.cn/myAnalysis.php) [51]. Odds ratios (ORs) and 95% confidence intervals (95% CIs) were calculated. The Hardy-Weinberg equilibrium (HWE) was evaluated applying the chi-square (*χ*^2^) test. A two-tailed *P* value less than 0.05 was regarded as statistically significant. All above-mentioned statistical analyses were performed by the SPSS 10.01 (SPSS Inc., IL, USA).

## 3. Results

### 3.1. Basic Characters of Study Subjects for Genotyping

The RA patients were composed of 101 males and 494 females with an average age of52.35 ± 12.75 years, while the healthy controls were composed of 100 males and 499 females with an average age of51.69 ± 6.83 years. No significant differences were found in age and gender between the RA patients and health controls. According to the data of anti-CCP and RF status, the RA patients can be divided into different serotypes. Finally, 487 (86.81%) patients were diagnosed as anti-CCP positive and 479 (83.16%) patients were diagnosed as RF positive (Table 1). The genotype distributions of rs10244329, rs2071045, and rs2167270 in healthy controls were in accordance with the Hardy-Weinberg equilibrium (all *P* > 0.05).

### 3.2. The Associations between *Leptin* Gene rs10244329, rs2071045, and rs2167270 Polymorphisms and RA Susceptibility

The genotype and allele frequencies of rs10244329, rs2071045, and rs2167270 in RA patients and healthy controls are expressed in Table 2. No significant difference in genotype distributions of three SNP polymorphisms was discovered in RA patients compared to healthy controls (all *P* > 0.05). Unfortunately, the three SNP polymorphisms also did not achieve a significant difference in allele frequencies between RA patients and controls (all *P* > 0.05). Moreover, there was no significant difference in the frequency of three SNP polymorphisms under two main genetic models (dominant and recessive models) between RA patients and controls (all *P* > 0.05).

### 3.3. The Associations of *Leptin* Gene rs10244329, rs2071045, and rs2167270 Polymorphisms with Risk of Different Serotypes of RA

A case-only study was conducted to analyze the relationship of *leptin* gene rs10244329, rs2071045, and rs2167270 polymorphisms and genetic susceptibility to different serotypes of RA patients. However, no significant genetic heterogeneity was observed between anti-CCP-positive and anti-CCP-negative RA patients and also between RF-positive and RF-negative RA patients (Table 3).

### 3.4. Haplotype Analysis

Eight haplotypes (AAC, AAT, ATC, ATT, GAC, GAT, GTC, and GTT) for *leptin* genes were identified by applying the SHEsis software (Table 4). Unfortunately, no significant differences in haplotype distributions of these three SNP polymorphisms between RA patients and healthy controls were observed (all *P* > 0.05).

### 3.5. Plasma Leptin Detection

We recruited 103 RA patients and 95 healthy controls for plasma *leptin* detection, without difference in gender and age. As per the results, no significant difference was found in plasma *leptin* concentrations between RA patients (10.90 ± 7.80 ng/ml) and healthy controls (12.36 ± 6.98 ng/ml) (*P* > 0.05) (Table 5). Further, in subgroup analyses, results showed no difference in plasma leptin levels between anti-CCP-/RF-positive and anti-CCP-/RF-negative groups of RA patients (Table 6).

## 4. Discussion

Rheumatoid arthritis is a chronic, complex autoimmune disease characterized by thickening and hyperplasia of the synovial line alongside permanent inflammation, causing severe disability and early death [3]. Although the exact pathogenesis of RA remains obscure, it may be associated with T cell immune responses and B cell-induced antibodies. In addition, cytokines from various immune cells play important roles in the pathogenesis of RA [20]. Increasing studies have confirmed that leptin plays important roles in inflammation and autoimmunity including RA [13–19]. Numerous studies have found significantly elevated serum levels of leptin in RA patients [27–35]. Nevertheless, results from these studies were inconsistent, and several studies had suggested decreased serum levels of leptin in RA patients [38–41]. Although the relationship is complex, emerging evidence has pointed out that leptin has been implicated in RA [7, 9, 20–26].

The role of *leptin* gene in autoimmune diseases has received much attention in recent years [42–46]. Farrokhi et al. discovered a notable difference in allele/genotype frequencies of the *leptin* gene among MS patients and health controls suggesting that *leptin* plays a crucial role in the susceptibility to MS and its severity [42]. Yousefian et al. also revealed that *leptin* polymorphisms were related to the risk of MS and its clinical symptoms [43]. Most recently, Chen et al. established a transgenic *leptin* pig overexpressing leptin and observed manifestations of SLE in this transgenic pig strain [44]. But a previous study of our group has not discovered the association between *leptin* polymorphisms with SLE in a Chinese population [45], which is consistent with previous studies [46].

It also has been reported that *leptin*-deficient mice had decreased levels of TNF-*α* and IL-1*β*, a less severe arthritis induced by antigen and defective cell-mediated immunity [47], whereas the reduction of leptin levels owing to fasting can lead to an improvement of clinical symptoms in RA patients [52]. Garcia-Bermudez et al. have assessed the relationships between *leptin* rs2167270 (19 G>A) gene polymorphism and the susceptibility to RA and have not found the association between *leptin* rs2167270 polymorphism and susceptibility to RA [48]. Afterward, Ali et al. have evaluated the association of *leptin* gene D7S1875 polymorphism with the risk for developing RA and severity of joint damage in patients with RA. The study concluded that the short allele of the D7S1875 (*leptin* gene) marker increases the risk for developing RA, but no association was observed between its allele/genotypes and RA [49].

Above all, we supposed that *leptin* gene polymorphism plays a critical role in the pathogenesis of RA. However, in this study, we did not discover any significant relationships between *leptin* gene polymorphism and RA. The three SNP polymorphisms did not achieve a significant difference in either genotype distributions or allele frequencies between RA patients and controls. In addition, no significant difference in haplotype distributions of these three SNP polymorphisms between RA patients and healthy controls was observed. Moreover, there was no significant difference of the three SNP polymorphisms under two main genetic models (dominant and recessive models) between RA patients and controls. Furthermore, no difference was detected in plasma leptin concentrations between RA patients and controls, and no association was shown between plasma leptin levels with clinical features of RA patients.

Finally, *leptin* rs10244329, rs2071045, and rs2167270 polymorphisms were shown to not be associated with susceptibility to RA. However, since this is not an overall study, the possible role of *leptin* in RA cannot be completely excluded. Some of the limitations in our study may affect the accuracy of the results, such as ethnic background, sample size, BMI, and patients with different disease activities, duration, and treatment. In addition, we cannot rule out that other polymorphisms in the *leptin* gene may be associated with RA. Therefore, it is also necessary to conduct repeated studies with larger sample sizes in different populations in the future.

## Figures and Tables

**Table 1:** The demographic characteristics and laboratory parameters of RA patients.

Characteristics	RA patients (*N* = 595)	Healthy controls (*N* = 599)
Age (years) mean ± SD	52.35 ± 12.75	51.69 ± 6.83
Sex (female/male)	494/101	499/100
Anti-CCP positive no. (%)	487 (86.81)	—
RF positive no. (%)	479 (83.16)	—

Anti-CCP: anticyclic citrullinated peptide; *N*: number; RA: rheumatoid arthritis; RF: rheumatoid factor.

**Table 2:** Genotype and allele frequencies of *leptin* gene in RA patients and controls.

SNP	Analyzed model	RA patients [*n* (%)]	Control [*n* (%)]	*P* value	OR (95% CI)
rs10244329	Genotype				
	AA	332 (55.80)	341(56.93)	0.255	0.765 (0.481-1.215)
	AT	228 (38.32)	211(35.23)	0.689	0.689 (0.428-1.109)
	TT	35 (5.88)	47(7.85)	Reference	
	Allele				
	A	892 (74.96)	893 (74.54)	0.815	0.978 (0.813-1.177)
	T	298 (25.04)	305(25.46)	Reference	
	Dominant model				
	AA	332 (55.80)	341 (56.93)	0.694	1.047 (0.833-1.316)
	TT+AT	263 (44.20)	258 (43.07)	Reference	
	Recessive model				
	AA+AT	560 (94.12)	552 (92.15)	0.180	0.734 (0.467-1.155)
	TT	35 (5.88)	47 (7.85)	Reference	
rs2071045	Genotype				
	CC	194 (32.61)	203 (33.89)	0.992	1.002 (0.723-1.387)
	CT	289 (48.57)	279 (46.58)	0.614	0.924 (0.680-1.256)
	TT	112 (18.82)	117 (19.53)	Reference	
	Allele				
	C	677 (56.89)	685 (57.18)	0.887	1.012 (0.860-1.190)
	T	513 (43.11)	513 (42.82)	Reference	
	Dominant model				
	CC	194 (32.61)	203 (33.89)	0.638	1.060 (0.833-1.348)
	TT+CT	401 (67.39)	396 (66.11)	Reference	
	Recessive model				
	CC+CT	483 (81.18)	482 (80.47)	0.756	1.047 (0.785-1.396)
	TT	112 (18.82)	117 (19.53)	Reference	
rs2167270	Genotype				
	GG	366 (61.51)	374 (62.44)	0.285	0.743 (0.431-1.282)
	GA	205 (34.45)	192 (32.05)	0.178	0.681 (0.389-1.194)
	AA	24 (4.03)	33 (5.51)	Reference	
	Allele				
	G	937 (78.74)	940 (78.46)	0.870	0.984 (0.809-1.196)
	A	253 (21.26)	258 (21.54)	Reference	
	Dominant model				
	GG	366 (61.51)	374 (62.44)	0.742	1.040 (0.823-1.314)
	AA+GA	229 (38.49)	225 (37.56)	Reference	
	Recessive model				
	GG+GA	571 (96.67)	566 (94.49)	0.232	0.721 (0.412-1.235)
	AA	24 (4.03)	33 (5.51)	Reference	

CI: confidence interval; OR: odds ratios; *n*: number; SNP: single nucleotide polymorphism.

**Table 3:** Associations of *leptin* gene polymorphisms with risk of different serotypes of RA.

SNP	Clinical features		Genotype [*n* (%)]			*P* value	Allele [*n* (%)]		*P* value
Allele (M/m)		Group	MM	Mm	mm		M	m	
rs10244329 (A/T)	Anti-CCP	Positive	270 (55.44)	186 (38.19)	31 (6.37)	0.583	726 (74.54)	248 (25.46)	0.314
		Negative	45 (60.81)	26 (35.14)	3 (4.05)		116 (78.38)	32 (21.62)	
	RF	Positive	262 (54.60)	187 (39.04)	30 (6.26)	0.319	711 (74.22)	247 (25.78)	0.129
		Negative	61 (62.89)	32 (32.99)	4 (4.12)		154 (79.38)	40 (20.62)	
rs2071045 (C/T)	Anti-CCP	Positive	159 (32.65)	232 (47.64)	96 (19.71)	0.215	550 (56.47)	424 (43.53)	0.825
		Negative	21 (28.38)	43 (58.11)	10 (13.51)		85 (57.43)	63 (42.57)	
	RF	Positive	153 (31.94)	232 (48.43)	94 (19.63)	0.607	538 (56.16)	420 (43.84)	0.351
		Negative	34 (35.05)	48 (49.48)	15 (15.46)		116 (59.79)	78 (40.21)	
rs2167270 (G/A)	Anti-CCP	Positive	294 (60.37)	173 (35.52)	20 (4.11)	0.973	761 (78.13)	213 (21.87)	0.234
		Negative	52 (70.27)	18 (24.32)	4 (5.41)		122 (82.43)	26 (17.57)	
	RF	Positive	290 (60.54)	168 (35.07)	21 (4.39)	0.373	748 (78.08)	210 (21.92)	0.179
		Negative	66 (68.04)	28 (28.87)	3 (3.09)		160 (82.47)	34 (17.53)	

M: major alleles; m: minor alleles; *n*: number; SNP: single nucleotide polymorphism.

**Table 4:** Haplotype analysis of SNPs in *leptin* gene in RA patients and controls.

Haplotype	Case [*n* (%)]	Control [*n* (%)]	*χ* ^2^	*P* value	OR (95% CI)
rs2167270-rs10244329-rs2071045					
AAC	2.41 (0.002)	1.50 (0.001)	—	—	—
AAT	1.77 (0.001)	0.59 (0.000)	—	—	—
ATC	5.19 (0.004)	7.48 (0.006)	—	—	—
ATT	243.63 (0.205)	248.44 (0.207)	0.030	0.862	0.983(0.806-1.198)
GAC	666.07 (0.560)	671.51 (0.561)	0.005	0.944	0.994 (0.845-1.170)
GAT	221.75 (0.186)	219.41 (0.183)	0.036	0.850	1.020 (0.829-1.255)
GTC	3.33 (0.003)	4.52 (0.004)	—	—	—
GTT	45.85 (0.039)	44.56 (0.037)	0.028	0.868	1.036 (0.681-1.578)

CI: confidence interval; OR: odds ratios; *n*: number.

**Table 5:** Characteristics of subjects included in plasma leptin level analysis.

Characteristics	RA patients (*N* = 103)	Healthy controls (*N* = 95)	*P* value
Age (years) mean ± SD	52.68 ± 11.98	51.08 ± 9.27	0.305
Sex (female/male)	90/13	80/15	0.523
Anti-CCP positive no. (%)	28 (84.8)	—	—
RF positive no. (%)	49 (84.5)	—	—
Leptin levels (ng/ml) mean ± SD	10.90 ± 7.80	12.36 ± 6.98	0.17

**Table 6:** Associations of plasma leptin levels and clinical features of RA patients.

Clinical features	Group	Leptin levels (ng/ml) mean ± SD	*P* value
Anti-CCP	Positive	10.43 ± 7.74	0.745
	Negative	9.19 ± 8.08	
RF	Positive	11.12 ± 8.03	0.284
	Negative	7.99 ± 7.51	

## Data Availability

The data are available upon request.
